# *β*-Catenin Expression Negatively Correlates with WIF1 and Predicts Poor Clinical Outcomes in Patients with Cervical Cancer

**DOI:** 10.1155/2016/4923903

**Published:** 2016-10-24

**Authors:** Jinxiao Liang, Hui Zhou, Yongpai Peng, Xiaofei Xie, Ruixin Li, Yunyun Liu, Qingsheng Xie, Zhongqiu Lin

**Affiliations:** ^1^Department of Gynecologic Oncology, Sun Yat-Sen Memorial Hospital, Sun Yat-Sen University, Guangzhou, China; ^2^Key Laboratory of Malignant Tumor Gene Regulation and Target Therapy of Guangdong Higher Education Institutes, Sun Yat-Sen University, Guangzhou, China

## Abstract

Aberrant activation of the canonical Wnt pathway plays a significant role in cervical cancer (CC). However, limited data show the correlation between the cancer clinicopathological characteristics and the key molecules such as *β*-catenin and Wnt inhibitory factor 1 (WIF1). In this study, *β-catenin* and* WIF1* expression were analyzed by immunohistochemistry for 196 patients with CC, 39 with cervical intraepithelial neoplasia (CIN), and 41 with normal cervical epithelium (NCE). Significant overexpression of *β-catenin* was detected in CC (67.9%) when compared to CIN (43.6%) or NCE (34.1%), *p* < 0.01, while low* WIF1* expression was detected in CC (24.0%) when compared to CIN (59.0%) or NCE (58.5%), *p* < 0.001. Negative correlation was shown between *β-catenin* and* WIF1* expression (*r* = −0.637, *p* < 0.001). In addition, multivariate analysis revealed that both lymph node metastasis and *β-catenin* expression were the independent prognostic factors not only for disease-free survival (HR = 5.029, *p* < 0.001; HR = 2.588, *p* = 0.035, resp.), but also for overall survival (HR = 5.058, *p* < 0.001; HR = 2.873, *p* = 0.031, resp.). Our findings indicate that, besides lymph node metastasis, *β*-catenin expression may also be a poor prognostic factor for CC while WIF1 could be a potential drug target for treatment of advanced CC.

## 1. Introduction

Cervical cancer (CC) is the fourth most common cancer in women. According to existing data, there were 528,000 registered new cases and 266,000 deaths only in year 2012 [1]. Most patients with early CC have good prognosis. By contrast, patients with a later cancer stage or metastatic CC have poor survival rate because of less effective treatments available [2]. Therefore, additional studies on late cancer development and prognosis methods are necessary.

Aberrant activation of the Wingless-type (Wnt)/*β*-catenin (canonical Wnt pathway) is a very common pathway in human CC [3]. Recent molecular testing has demonstrated that the CC biological behavior may arise as a multistep gene process. Specifically, infection with human papillomavirus (HPV) could be “the first hit” [4], while the dysregulation of canonical Wnt pathway may be required as “the second hit” in cervical oncogenesis [3, 5, 6]. However, the mechanism involving Wnt pathway in CC is still not well understood and requires additional studies. In the canonical Wnt pathway, the binding of Wnts to a heterodimeric receptor complex stabilizes the *β-catenin* expression and leads to the activation of *β-catenin* target genes inside the nucleus [7, 8]. Furthermore, Wnt inhibitory factor 1 (*WIF1*) is an upstream secreted Wnt antagonist, first identified as highly conserved gene in the human retina [9].* WIF1* main function is to bind the extracellular Wnt ligands [10], disturbing the Wnt interaction with the receptors [7] and consequently leading to *β-catenin* degradation, therefore inhibiting the canonical Wnt pathway. Currently, there are dozens of studies regarding the correlation with *β-catenin* and various types of cancer [7, 11]. In most of these cases, elevated levels of *β*-catenin have been strongly correlated with poor cancer prognosis. However, so far, there have been limited reports focusing on the association between *β-catenin*,* WIF1*, and the clinicopathological characteristics in CC.

In the present study, we investigated the association between the canonical Wnt pathway (*β-catenin* as the hallmark [7]),* WIF1*, and clinicopathological features of 196 patients with CC and analyzed their prognostic value in CC.

## 2. Materials and Methods

### 2.1. Patients and Tissue Samples

Between December 2002 and October 2007, 196 patients from the Department of Gynecologic Oncology (Sun Yat-Sen Memorial Hospital, Canton, China), diagnosed with CC (stages IA–IIB) and submitted to curative surgical resection, were recruited for this study. Cases were restricted to those who did not receive any tumor related treatment before surgery. The clinicopathological characteristics were summarized in Table 3. Furthermore, an additional 39 cervical intraepithelial neoplasia (CIN) and 41 normal cervical epithelium (NCE) samples were obtained from women undergoing hysterectomy for noncancerous diagnosis. Histological diagnosis and tumor stage and grade were determined according to the World Health Organization (WHO) and the International Federation of Gynecology and Obstetrics (FIGO) staging systems [12, 13]. All specimens were anonymously coded in accordance with local ethical guidelines (as stipulated by the Declaration of Helsinki). All study protocols were approved by the University Ethics Committee.

### 2.2. Immunohistochemistry (IHC) and Samples Evaluation

According to the previously described method [14, 15], IHC analysis was performed using anti-*β*-catenin antibody (CST, #9562, Boston, MA, America) and anti-WIF1 antibody-N-terminal (Abcam, ab71204, Cambridge, MA, America). Human breast tissues were used as positive controls; negative controls were obtained by replacing the primary antibodies with phosphate buffered saline. The staining of *β*-catenin was evaluated according to Maruyama's method [16]. If more than 10% of cancer cells were positively stained for cytoplasm and/or nuclei, the cells were regarded as *β*-catenin-positive expression. By contrast, membrane staining was only classified as *β*-catenin-negative expression; for WIF1 protein expression, nuclear staining was considered to be negative [17]. Finally, if more than 10% of cancer cells were positively stained for WIF1 in cytoplasmic and/or on cell membranes, the positive IHC results were recorded.

### 2.3. Statistical Analysis

All the statistical analysis was conducted using SPSS software, version 13.0 (SPSS Inc., Chicago, IL, USA). Associations between the clinicopathological characteristics and the pattern of WIF1 and *β*-catenin expression were examined using Pearson's *χ* ^2^ test. Survival rates were calculated using the Kaplan-Meier method and compared using the log-rank test. Univariate and multivariate survival analyses were performed using the Cox regression model for DFS and OS. A forward stepwise procedure was used to identify independent variables in the multivariate analysis. *p* < 0.05 indicated statistical significance.

## 3. Results

### 3.1. IHC of *β*-Catenin and WIF1 and Their Correlation with CC

**Figure 1:**
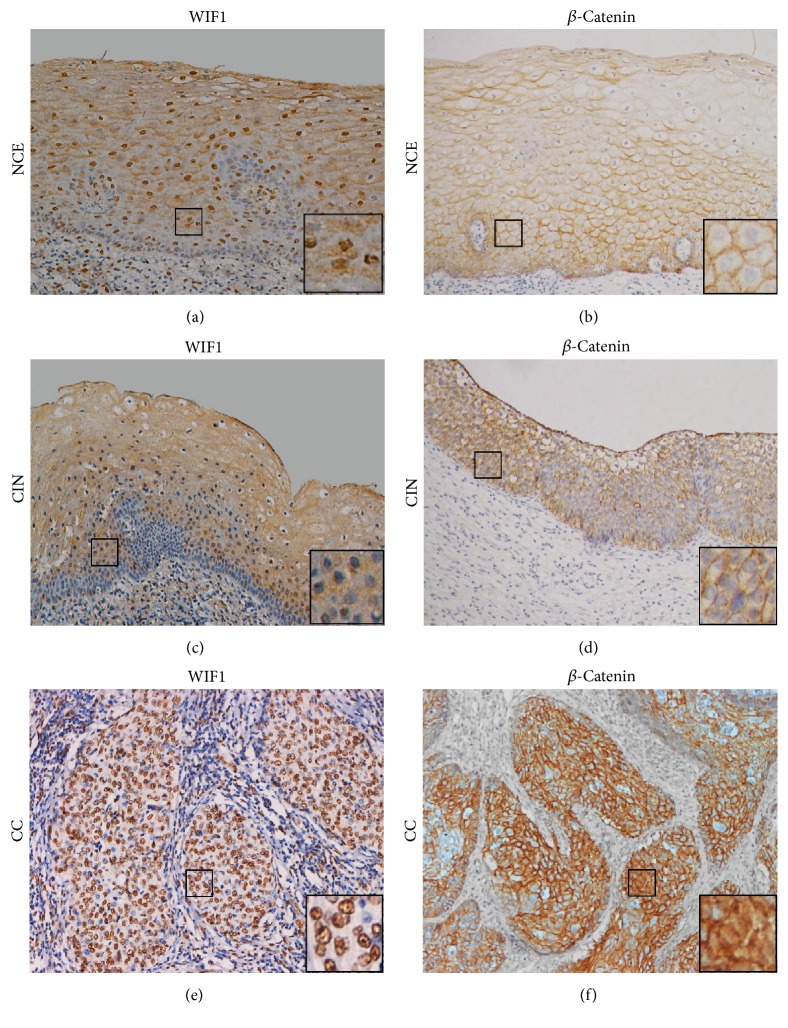
WIF1 and *β*-catenin staining images of human cervical tissues by IHC. ((a), (c)) Positive cytoplasmic staining of WIF1 was observed in both NCE and CIN. ((b), (d)) Positive membranous staining with no cytoplasmic/nuclear staining of *β*-catenin was observed in both NCE and CIN. (e) Positive nuclear staining without membranous/cytoplasmic staining of WIF1 was observed in CC. (f) Positive cytoplasmic staining of *β*-catenin was observed in CC. Magnification: ×200 (hematoxylin counterstained). Insets are magnified images from selected areas (small squares).

As shown in Figure 1 and Table 1, obvious membranous staining of *β*-catenin and nuclear staining of WIF1 were observed in most cancer and noncancerous samples; however, both staining patterns were considered to be negatively expressed. No significant difference was observed between *β*-catenin-positive expression ratios (cytoplasmic/nuclear staining) in CIN (17/39, 43.6%) compared to NCE (14/41, 34.1%). In addition, positive *β*-catenin expression was found in (133/196, 67.9%) cervical cancer samples (both with *p* < 0.01). Furthermore, low expression of WIF1 was significant in CC samples compared with CIN and NCE (*p* < 0.001). Briefly, from 149 WIF1 negative CC samples, 126 (84.6%) were *β*-catenin-positive. On the other hand, from 47 WIF1 positive CC samples, 40 (85.1%) were *β*-catenin-negative (*p* < 0.001), Table 2. Statistically, *β*-catenin expression had negative correlation compared to WIF1 expression (*r* = −0.637; *p* < 0.001).

### 3.2. IHC of *β*-Catenin and WIF1 in Representative Cases with CC

**Figure 2:**
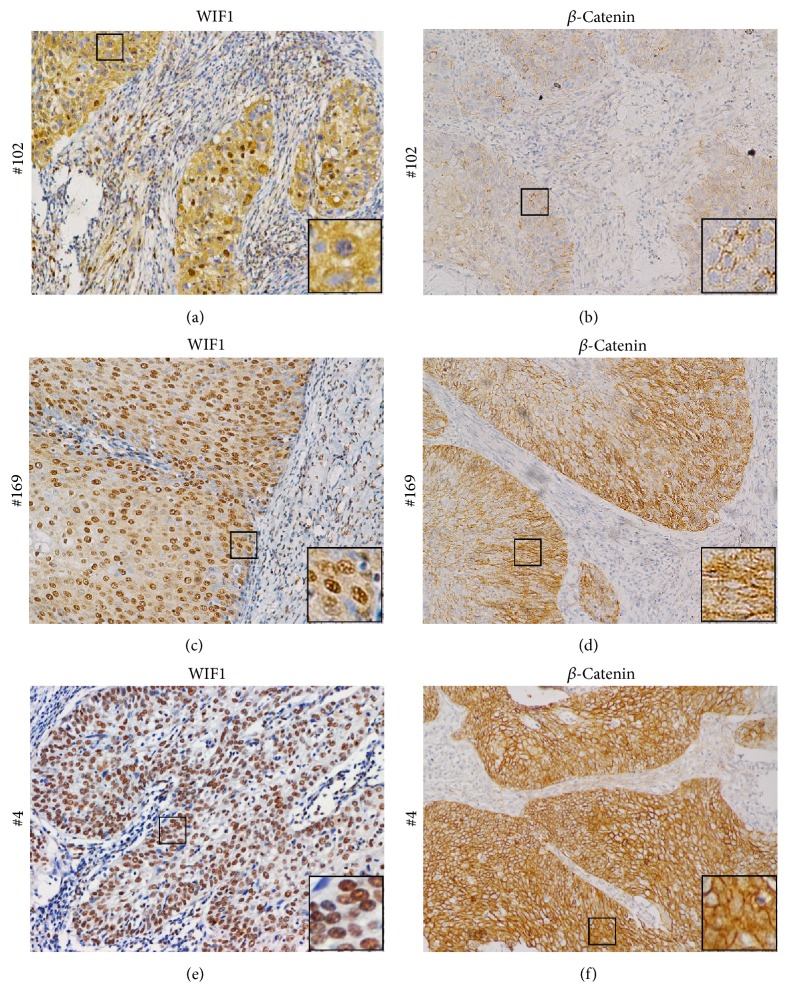
Comparison of WIF1 and *β*-catenin expression in CC. The relationship of WIF1 and *β*-catenin immunostaining for three representative cases: ((a), (b)) Case  102, indicating strong WIF1 cytoplasmic staining and negative *β*-catenin cytoplasmic/nuclear staining; ((c), (d)) Case  169, which shows moderate immunostaining for both WIF1 and *β*-catenin; ((e), (f)) Case  4, showing negative WIF1 cytoplasmic staining and strong *β*-catenin cytoplasmic staining. Magnification: ×200. Insets are magnified images from selected areas (small squares).

Images of WIF1 and *β*-catenin staining for representative CC cases were shown in Figure 2, which indicated that, with the decreased expression of WIF1, clearly increased expression of *β*-catenin was observed. Specifically, the expression of *β*-catenin was usually absent in patients who had the most intense cytoplasmic WIF1 staining while the staining of *β*-catenin was intense in those who had no cytoplasmic expression of WIF1.

### 3.3. Special WIF1 Staining Pattern of CC Cells with Mitotic Figures

**Figure 3:**
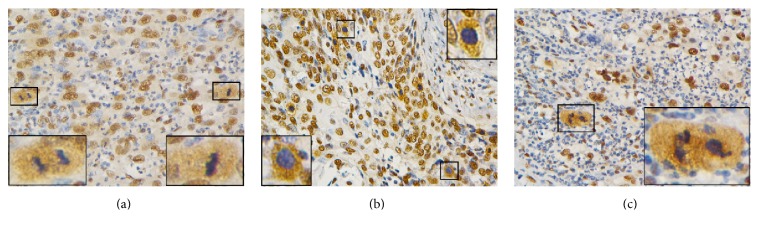
Pleomorphic WIF1 staining appearances of CC cells with mitotic figures. The blue-stained (hematoxylin stain) nuclei and brown-stained (WIF1 stain) cytoplasm were characteristic. (a) CC cells in metaphase with small short rod-like nuclei and medium-sized cytoplasm were shown on the right while CC cells in telophase with mirror image were seen on the left. (b) CC cells in prophase with round nuclei and intermediate-sized cytoplasm (sunflower-like appearance) were shown. (c) CC cells in triploid mitotic figures with apparently lobulated nuclei and abundant brown-stained cytoplasm were shown. Magnification: ×400. Insets are magnified images from selected areas (small squares).

WIF1 was usually found to be moderate-to-strong nuclear staining but without cytoplasmic staining in this study. However, as shown in Figure 3, another pattern of WIF1 staining was observed in mitotic figures in some CC samples, which were characterized by blue-staining (hematoxylin stain) nuclei and brown-staining (anti-WIF1 stain) cytoplasm and were registered for the first time. The individual mitotic figures could present pleomorphic appearances, such as mirror-image cells (Figure 3(a)) and sunflower-like cells (Figure 3(b)).

### 3.4. The Expression of WIF1 and *β*-Catenin and Their Association with the Clinicopathological Features of CC

As shown in Table 3, *β*-catenin-positive expression was associated with a higher rate of lymphovascular space invasion (*p* = 0.017). Furthermore, WIF1 positive staining was associated with less cervical stromal invasion (*p* = 0.002) and a lower rate of lymphovascular space invasion (*p* = 0.035).

### 3.5. The Clinicopathological Features and Their Prognostic Values

**Figure 4:**
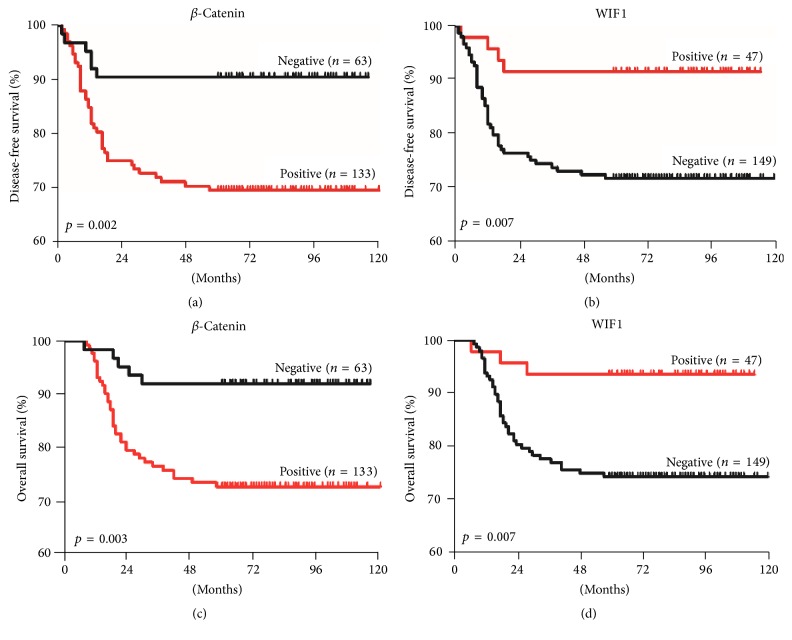
Kaplan-Meier 5-year disease-free survival (DFS) and 5-year overall survival (OS) curves for patients with cervical cancer. ((a) and (c)) Patients with *β*-catenin-positive tumors tended to have poorer DFS and OS. ((b) and (d)) Patients with WIF1-positive expression had significantly better DFS and OS than those with negative results.

During the median follow-up of 70 months (range: 60–121 months), 46/196 (23.5%) patients underwent CC recurrence. Among those patients, 41 (88.1%) died from cancer progression and 5 patients with recurrent vaginal or lymph node CC survived after surgery and adjuvant concurrent chemoradiotherapy. The 5-year disease-free survival (DFS) and overall survival (OS) rates were 76.5% and 79.1%, respectively. CC patients with positive *β*-catenin expression had poorer 5-year DFS (69.9% versus 90.5%, *p* = 0.002; Figure 4(a)) and 5-year OS (72.9% versus 92.1%, *p* = 0.003; Figure 4(c)) than patients with negative *β*-catenin expression. Patients with positive WIF1 expression had longer 5-year DFS (91.5% versus 71.8%, *p* = 0.007; Figure 4(b)) and OS (93.6% versus 74.5%, *p* = 0.007; Figure 4(d)) than those with negative WIF1 results.

The univariate analysis showed that advanced FIGO stage, parametrial invasion, positive surgical margin, lymph node metastasis, larger tumor size (>4 cm), and *β*-catenin expression were correlated with poorer 5-year DFS rate. In addition, lymph node metastasis (*p* < 0.001; hazard ratio (HR) = 5.029; 95% CI: 2.623–9.645) and *β*-catenin expression (*p* = 0.035; HR = 2.588; 95% CI: 1.071–6.251) emerged as independent predictors of 5-year DFS in multivariate analysis, Table 4. For 5-year OS, FIGO stage, parametrial invasion, positive surgical margin, lymph node metastasis, larger tumor size, and *β*-catenin expression were included in the multivariate analysis. Lymph node metastasis (*p* < 0.001; HR = 5.058; 95% CI: 2.524–10.137) and *β*-catenin expression (*p* = 0.031; HR = 2.873; 95% CI: 1.102–7.492) emerged as independent predictors of 5-year OS, Table 4.

## 4. Discussion

Dysregulation of* Wnt *pathway is involved in different diseases, including cancer. *β-Catenin*, the key factor of canonical* Wnt* pathway, conducts* Wnt *signals to the nucleus and upregulates oncogenes during tumorigenesis [7, 8]. It has been demonstrated that the expression of *β-catenin *can be upregulated in various cancers, including CC [3, 5, 18]. Consistent with previous reports [19–21], *β-catenin* accumulation inside the cytoplasm was found to be significantly increased in CC when compared with CIN and NCE in this study. In addition, by Cox regression analysis, it was shown that *β-catenin*-positive expression was significantly correlated with poor prognosis in 5-year DFS and 5-year OS. In the present study, a negative correlation between cytoplasmic/nuclear *β-catenin* accumulation and cytoplasmic* WIF1* immunostaining (positive expression) was found. In addition, decreased* WIF1* expression in CC was consistent with some previous studies which were based on human tumor study, such as gastrointestinal tract, kidney, glioblastoma, osteosarcoma, lung, pituitary, bladder, and oral cavity [17, 22–28]. Therefore, we hypothesize that the canonical* Wnt* pathway was activated, whereas the* Wnt* antagonist* WIF1 *was inhibited, by the multistep gene process in CC.

Our results have suggested that WIF1 expression was negative in 94.7% of CC samples with *β-catenin*-positive expression (Table 2), while the results in Figure 2 support the association between downregulation of WIF1 and upregulation of *β*-catenin expression in CC. This may indicate that inactivation of WIF1 and accumulation of stabilized *β-catenin* are a gradual process during tumorigenesis and progression of CC. Our study showed that the positive staining of* WIF1* was significantly reduced in patients with >1/2 cervical stromal invasion and lymphovascular space invasion, while positive staining of *β-catenin* was associated with lymphovascular space invasion. Furthermore, though no statistical significance was registered, the expression of* WIF1 *was much lower in the patients with surgical margin involvement (14.3%) and lymph node metastasis (14.8%) than in those with no surgical margin involvement (24.3%) or no lymph node metastasis (27.5%; Table 3). These results stand in support of the idea that* WIF1* downregulation is an early event [6] and can potentially inhibit the early progression stage of CC by antagonizing canonical* Wnt *pathway.

Recurrence in many advanced cancers has been associated with chemoresistance. The related mechanisms include tumor angiogenesis, maintenance of resistant cancer stem cells, dysregulation of cell cycle, and defects in apoptosis, which are all at least partly regulated by the canonical Wnt pathway [29]. The expressions of components related to this pathway are frequently altered. Increased expression of Wnt ligands was reported in breast cancer [30] and dishevelled in cervical cancer [31], while decreased expression of dickkopf-1 was reported in pancreatic cancer [32], secreted frizzled-related protein 1 (SFRP1) in lung cancer [33], and WIF1 in cervical cancer [6]. The potential therapeutic targets related to these compounds in cancer have been investigated through successful preclinical studies [34]. Therefore, just as in some other carcinomas [22, 35–37],* WIF1* is a potent drug target in CC treatment.

Although the cytoplasmic* WIF1* staining pattern was consistent with previous studies [6, 14, 15], the moderate-to-strong brown nuclear staining of* WIF1* was common in our study. In some CC cases, mitotic figures were cytoplasm-positive and nuclei-negative staining (Figure 3), which made them easily identifiable compared to nonmitotic cancer cells. These results indicate that the changes of the localization of WIF1 expression may be related to the uncontrolled CC cell division. However, at present, the reason of opposite cytoplasmic/nuclear WIF1 staining patterns between mitotic figures and nonmitotic CC cells is unclear. Lack of study on the mechanisms and functions of the translocation of WIF1 between mitotic figures and nonmitotic CC cells is a limitation of our study. Further investigations for the reason are needed.

## 5. Conclusions

Our results have demonstrated the upregulation of *β-catenin *and downregulation of* WIF1* in CC samples compared to CIN and NCE. Along with clinicopathological characteristics, such as lymph node metastasis and cervical stromal invasion, increased *β-catenin* expression has been shown to be a poor prognostic factor for CC, indicating the aberrant activation of canonical Wnt pathway. Moreover, the WIF1 staining pattern in mitotic figures (IHC, cytoplasm-positive and nucleus-negative) was opposite to most other CC cells in this study, and the reason needs to be explored in the future.

## Figures and Tables

**Table 1:** Comparison of WIF1 and *β*-catenin expression in NCE, CIN, and CC.

Variable	Cases (number)	*β*-Catenin			WIF1		
		Negative	Positive	*p* ^a^	Negative	Positive	*p* ^a^
CC	196	63	133 (67.9%)	<0.01^b^	149	47 (24.0%)	<0.001^c^
CIN	39	22	17 (43.6%)		16	23 (59.0%)	
NCE	41	27	14 (34.1%)		17	24 (58.5%)	

^a^The *p* value was determined using the *χ* ^2^ test. ^b^ *p* < 0.01 was found in the ratio of *β*-catenin positive expression when compared to CC and CIN or CC and NCE; ^c^ *p* < 0.001 was found in the ratio of WIF1 positive expression when compared to CC and CIN or CC and NCE; no significant differences were observed when comparing CIN and NCE.

**Table 2:** The relationship of WIF1 and *β*-catenin in CC.

Variable	WIF1		*p* ^a^	Correlation coefficient
	(−)	(+)		
*β*-Catenin				
(−)	23	40	***<*0.001**	−0.637
(+)	126	7		

^a^The *p* value was determined using the *χ* ^2^ test.

**Table 3:** Association between the expression of WIF1 and *β*-catenin and clinicopathological parameters of CC (stages IA–IIB).

Variable	Cases		WIF1			*β*-Catenin		
	Number	(%)	Negative	Positive	*p* ^a^	Negative	Positive	*p* ^a^
Histologic subtype								
Squamous cell carcinoma	172	87.8	133	39	NS	58	114	NS
Adenocarcinoma	24	12.2	16	8		5	19	
FIGO stage								
IA	16	8.2	13	3	NS	6	10	NS
IB1, IIA1	102	52.0	77	25		31	71	
IB2, IIA2	64	32.7	46	18		25	39	
IIB	14	7.1	13	1		1	13	
Tumor grade^*∗*^								
G1	35	19.2	27	8	NS	6	29	NS
G2/G3	147	80.8	112	35		41	106	
Parametrial invasion								
Negative	186	94.9	141	45	NS	62	124	NS
Positive	10	5.1	8	2		1	9	
Surgical margin involved								
No	189	96.4	143	46	NS	63	126	NS
Yes	7	3.6	6	1		0	7	
Lymph node metastasis								
Negative	142	72.4	103	39	NS	51	91	NS
Positive	54	27.6	46	8		12	42	
Tumor size (cm)								
≤4	117	59.7	90	27	NS	36	81	NS
>4	79	40.3	59	20		27	52	
Cervical stromal invasion								
≤ one second	86	43.9	56	30	**0.002**	27	59	NS
> one second	110	56.1	93	17		34	76	
Lymphovascular invasion^*∗*^								
Negative	93	61.6	64	29	**0.035**	33	60	**0.017**
Positive	58	38.4	49	9		10	48	
Age (years)								
≤35^*∗∗*^	35	17.9	30	5	NS	9	26	NS
>35	161	82.1	119	42		54	107	

NS: not significant; HPF: high-power field. ^a^The *p* value was determined using the *χ* ^2^ test. Significant *p* values are shown with bold font. ^*∗*^The number of patients is less than 196 because of missing data. ^*∗∗*^Age (range: 23–66 y, median: 42 y).

**Table 4:** Univariate and multivariate analysis of factors associated with DFS and OS for patients with CC.

Variable	5-year DFS^c^				5-year OS^c^			
	Univariate	Multivariate^d^			Univariate	Multivariate^d^		
	*p* ^a^	*p* ^a^	HR^b^	95% CI	*p* ^a^	*p* ^a^	HR^b^	95% CI
FIGO stage								
IA	<**0.001**	NS			**0.002**	NS		
IB1, IIA1								
IB2, IIA2								
IIB								
Parametrial invasion								
Negative	**0.002**	NS			**0.007**	NS		
Positive								
Surgical margin involved								
No	**0.008**	NS			**0.036**	NS		
Yes								
Lymph node metastasis								
Negative	<**0.001**	<**0.001**	5.029	2.623–9.645	<**0.001**	<**0.001**	5.058	2.524–10.137
Positive								
Tumor size (cm)								
≤4	<**0.001**	NS			**0.001**	NS		
>4								
*β*-Catenin								
Negative	**0.001**	**0.035**	2.588	1.071–6.251	**0.002**	**0.031**	2.873	1.102–7.492
Positive								

DFS: disease-free survival; OS: overall survival; HR: hazard ratio; CI: confidence interval; NS: not significant; HPF: high-power field. ^a^Significant *p* values are shown in bold font. ^b^HR > 1 indicates that risk for recurrence/death increased; HR < 1 indicates that risk for recurrence/death decreased. ^c^Univariate and multivariate analyses and Cox proportional hazards regression model. ^d^Variables associated with survival by univariate analysis were adopted as covariates in multivariate analyses.
